# Therapy-associated facial redness in atopic dermatitis: insights from a case series

**DOI:** 10.1016/j.abd.2026.501398

**Published:** 2026-06-22

**Authors:** Karol Sabas-Ortega, Sergio García-González, Mariano Ara-Martín, Ana Luisa Morales-Moya, Sonia de la Fuente Meira, Elena Bularca, Pablo Villagrasa-Boli, Luis Martinez-Lostao, Ignacio Rivera-Fuertes, Lucía Prieto-Torres

**Affiliations:** a Department of Dermatology, Hospital Clínico Universitario Lozano Blesa, Universidad de Zaragoza, Zaragoza, Spain; b Aragon Health Research Institute, GIIS100 Health Research Group, Zaragoza, Spain; c Department of Dermatology, Hospital Barbastro, Huesca, Spain; d Department of Immunology, Hospital Clínico Universitario Lozano Blesa, Universidad de Zaragoza, Zaragoza, Spain

Dear Editor,

Atopic Dermatitis (AD) is a chronic inflammatory disease that significantly impacts patients’ quality of life. Novel targeted biologics and small-molecule immune modulators have revolutionized its management. While these agents have transformed AD management, a new dermatological phenomenon ‒ Facial Redness (FR) ‒ has been increasingly reported, particularly with dupilumab. This condition lacks a standardized term and has been variably referred to in the literature as “dupilumab facial redness”, “dupilumab facial dermatitis”, and “paradoxical head and neck erythema,” among others. Although most cases are linked to dupilumab, two reports have also documented FR with tralokinumab^1^ and upadacitinib.^2^

We retrospectively reviewed 101 patients with moderate-to-severe AD under biologic or immunomodulatory therapy in our department and identified 11 patients (10.9%) who developed FR during treatment. Clinical and laboratory data were extracted from medical records, including total and *Malassezia*-specific Immunoglobulin E (IgE) levels. *Malassezia*-specific IgE was measured using the ImmunoCAP™ fluoroenzyme immunoassay (Thermo Fisher Scientific), code M227, a whole-extract *Malassezia* spp. preparation that includes antigens from multiple species. Cross-reactive carbohydrate determinant (CCD) sensitization was evaluated using MUXF3 (O214), and no CCD reactivity was detected, confirming that M227 results were not CCD-driven. Direct microscopy examination for *Demodex* and *Malassezia* was not performed. This method has shown limited sensitivity for *Demodex* (approximately 50%).^3^ Similarly, direct examination for *Malassezia*-related conditions shows reduced sensitivity, with low detection rates reported for *Malassezia* folliculitis (59%) and seborrheic dermatitis (35%), and high operator dependency.^4^

Patient and disease characteristics are summarized in Table 1. Most patients were female (91%), with a median age of 29-years. Notably, four patients (36%) experienced FR more than once with different therapeutic agents. Time to onset of FR was measured in weeks following the start of biologic or immunomodulatory therapy (Table 2), and no significant association between treatment and onset time was observed. Across the series, 8 patients had been exposed to dupilumab, 6 to tralokinumab, 3 to upadacitinib, 3 to abrocitinib, and 1 to lebrikizumab. A total of 8 FR episodes occurred during dupilumab therapy, 5 with tralokinumab, and single events with upadacitinib, abrocitinib, and lebrikizumab. A more detailed description of each patient can be observed in Fig. 1.

**Table 1:** Patient and disease characteristics of participants in this retrospective analysis.

	1	2	3	4^a^	5	6	7	8	9	10	11
**Sex**	Male	Female	Female	Female	Female	Female	Female	Female	Female	Female	Female
**Age**	42	29	27	29	51	22	20	35	32	89	18
**Disease onset**	Child	Teen	Child	Child	Adult	Child	Child	Teen	Child	Adult	Child
**Comorbidities**	Asthma, chronic urticaria	Asthma, allergic rhinoconjunctivitis, ACD	Allergic rhinoconjunctivitis	ACD	Chronic urticaria, allergic rhinoconjunctivitis, hypothyroidism	–	Obesity	Allergic rhinoconjunctivitis	–	–	Asthma, Food allergies
**Previous facial AD**	+	+	+	+	+	–	+	+	+	+	+
**Previous systemic immunosuppressants**	GCS, CsA	GCS, MTX	CsA, Abrocitinib	CsA	AZA, CsA, GCS	GCS, AZA, CsA, Baricitinib	GCS, CsA	GCS, AZA, CsA	GCS, CsA, Tralokinumab, Abrocitinib	GCS, MTX	GCS, CsA, Upadacitinib, Dupilumab
**Onset of red face (weeks since treatment)**	13 Dupilumab	28.2 Dupilumab	13 Tralokinumab	26 Tralokinumab	8.6 Tralokinumab	0.57 Dupilumab	4 Dupilumab	4 Tralokinumab	2.8 Dupilumab	35 Tralokinumab	8 Dupilumab
			21 Upadacitinib	0.42 Dupilumab	20 Dupilumab						8 Lebrikizumab
				13 Abrocitinib							
**Treatment Systemic**	Itraconazole 100 mg daily ×14 days	Itraconazole 100 mg daily ×14 days	Itraconazole 100 mg daily ×7 days, GCS	Itraconazole 100 mg daily ×14 days, switch Upadacitinib	Itraconazole 50 mg daily ×14 days	GCS	switch Upadacitinib	Azithromycin 500 mg × 3 days	–	GCS	Itraconazole 100 mg daily ×14 days + GCS
**Topical treatment**	TCI	TCI	–	TCI	–	TCI, clotrimazole	–	Mupirocin	TCI, clotrimazole	Fluticasone	Fluticasone
**Biopsy**	Spongiotic dermatitis	‒	–	–	–	–	–	–	–	–	–

ACD, Allergic Contact Dermatitis; GCS, Glucocorticoids; CsA, Ciclosporine; MTX, Methrotexate; AZA, Azathioprine; TCI, Topical Calcineurin Inhibitors.

Spongiotic dermatitis showing superficial lymphocytic infiltrate and sawtooth acanthosis along with spongiosis and exocytosis of lymphocytes compatible with atopic dermatitis.

a Previously published case report (1).

**Table 2:** Onset of facial redness (weeks of treatment).

				95% CI			
	Treatment	n	Mean	Lower	Upper	Median	IQR
**Onset of Facial Redness (Weeks)**	Abrocitinib	1	13.0	null	null	13.0	0.0
	Dupilumab	8	9.6	0.42	28.3	6.0	12.5
	Lebrikizumab	1	8.0	null	null	8.0	0.0
	Tralokinumab	5	17.3	4	35.0	13.0	17.4
	Upadacitinib	1	21.0	null	Null	21.0	0.0

**Figure 1:**
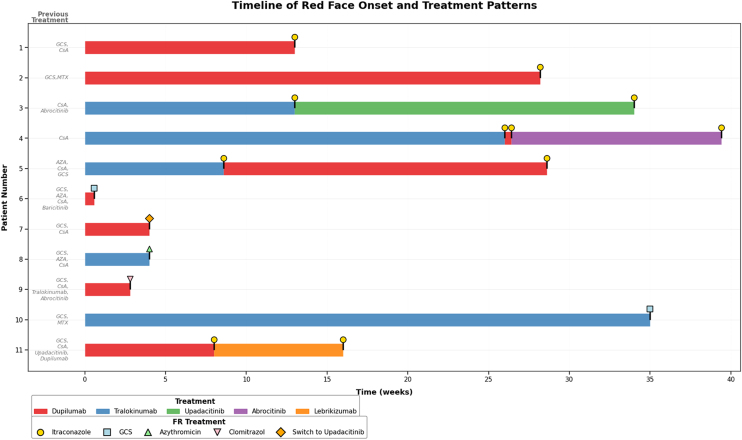
Timeline of FR events in our case series.

## IgE and *Malassezia*-specific IgE serum levels

Elevated total and *Malassezia*-specific IgE levels were found in 55% of patients. Those with increased *Malassezia*-specific IgE had higher odds of responding to antifungal therapy compared with those with normal values (OR = 7.5, 95% CI 1.03–54.9; p = 0.045).

**Figure 2:**
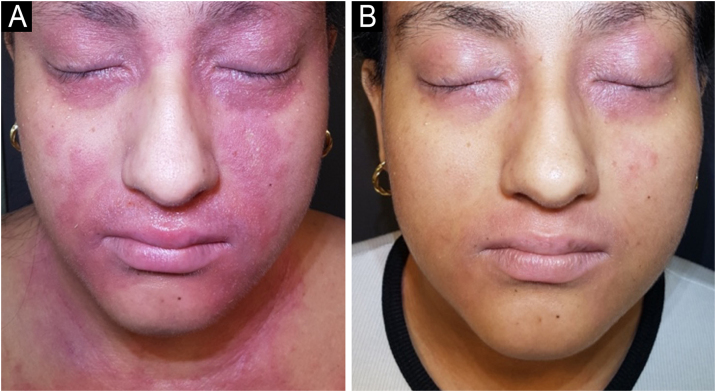
Before (A) and after (B) of Facial Redness (FR) associated to Lebrikizumab in case #11 (A‒B) after antifungal treatment.

Among the cases, patient 11 deserves particular attention, as it represents the first reported case of lebrikizumab-associated FR. She was an 18-year-old female with severe AD who experienced secondary failure to upadacitinib and was switched to dupilumab. Eight-weeks later, she developed a severe generalized AD outbreak with moderate FR. Due to a lack of symptom control, a switch to lebrikizumab was made. Eight weeks after the start of lebrikizumab, the patient developed a new flare of mainly facial eczema and eczematous plaques with scalp pustules (Fig. 2A). *Malassezia*-specific IgE was the highest in our series (69.5 KU/L) and therefore received itraconazole 100 mg daily for a month along with a short course of oral corticosteroids was initiated, resulting in great improvement in the FR (Fig. 2B). Lebrikizumab has been continued and no further therapeutic switch has been necessary to date. To our knowledge, this represents the first reported case of FR associated with lebrikizumab (Table 3).

**Table 3:** *Malassezia* specific IgE levels in our case series.

Patient	#1	#2	#3	#4	#5	#6	#7	#8	#9	#10	#11
IgE *Malassezia*	0.1 KU/L	0.1 KU/L	11.4 KU/L	3.31 KU/L	6.68 KU/L	0.1 KU/L	0.1 KU/L	11.2 KU/L	0.76 KU/L	<0.1 KU/L	69.5 KU/L
IgE *Malassezia* classification	Normal	Normal	High	High	High	Normal	Normal	High	High	Normal	High
Antifungal response	+	–	+	+	+	+	–	N/A	+	–	+

The development of FR during treatment with biologic or immunomodulatory agents represents a diagnostic and therapeutic challenge due to its multifactorial pathogenesis. Proposed mechanisms include: *Malassezia* hypersensitivity,^5^, ^6^ allergic contact dermatitis (ACD),^6^, ^7^ *Demodex folliculorum* overgrowth,^8^ topical corticosteroid withdrawal,^6^ and localized treatment failure.^7^

Our findings support the multifactorial nature of FR. Elevated *Malassezia*-specific IgE and antifungal response in several patients reinforce that prolonged suppression of Th2-driven inflammation may upregulate the Th17 pathway, promoting *Malassezia* proliferation, as previously described by other authors.^6^ ACD also remains an important differential diagnosis, as IL-4 inhibition may also enhance Th1/Th17 and promote allergen responses.^9^, ^10^ In our series, one patient also showed a positive patch test to nickel, which suggests ACD could be involved in the pathogenesis of her FR.

One patient improved with macrolides (Patient 8), consistent with *Demodex* overgrowth etiology, as previously described by Seok et. al.^11^ Experimental models with impaired Th2 function have shown an increase in *Demodex* infestation^11^ that suggests an involvement of Th1/Th17 upregulation.^7^, ^9^, ^12^

Most patients had a prior history of head and neck dermatitis, suggesting that localized treatment failure may also contribute to the pathogenesis. Particularly in recurrent FR episodes with multiple agents.

Given the probable multifactorial nature of paradoxical FR, we recommend baseline measurement of total and *Malassezia*-specific IgE prior to the start of AD systemic treatment and, as suggested by other authors, in patients with severe atopic dermatitis involving the face, patch testing should also be considered to rule out sensitization to contact allergens and airborne contact dermatitis. This approach may facilitate the identification of underlying mechanisms and help tailor treatment strategies to optimize patient outcomes.

Our series expands the scope beyond the recognized link to dupilumab to include tralokinumab, iJAKs, and notably the first case with lebrikizumab. These findings suggest that FR is likely due to a paradoxical inflammatory phenomenon secondary to immunemodulation rather than being drug-specific. Larger studies are needed to elucidate more of its pathogenesis and optimize management strategies.

## ORCID ID

Sergio García-González: 0009-0006-2628-092X

Mariano Ara-Martín: 0000-0001-8789-6783

Ana Luisa Morales-Moya: 0000-0002-4747-0152

Sonia de la Fuente Meira: 0000-0002-6328-4958

Elena Bularca: 0000-0001-9043-4985

Pablo Villagrasa-Boli: 0000-0003-2552-951X

Luis Martinez-Lostao: 0000-0003-3043-147X

Ignacio Rivera Fuertes: 0000-0001-9977-6615

Lucía Prieto-Torres: 0000-0002-5066-5014

## Research data availability

The entire dataset supporting the results of this study was published in this article.

## Financial support

None declared.

## Authors’ contributions

Karol Sabas Ortega: Study conception and planning; statistical analysis; preparation and writing of the manuscript.

Sergio Garcia Gonzalez: Data collection, analysis and interpretation.

Ana Luisa Morales Moya: Intellectual participation in propaedeutic and/or therapeutic management of studied cases.

Luis Martinez Lostao: Intellectual participation in propaedeutic and/or therapeutic management of studied cases.

Elena Bularca: Effective participation in research orientation.

Ignacio Rivera: Effective participation in research orientation.

Pablo Villagrasa: Effective participation in research orientation.

Sonia de la Fuente: Effective participation in research orientation.

Lucia Prieto Torres: Effective participation in research orientation; manuscript critical review; approval of the final version of the manuscript.

Mariano Ara Martin: Approval of the final version of the manuscript.

## Conflicts of interest

None declared.

## References

[1] García-González S., Villagrasa-Boli P., Bularca E., Moratiel-Pellitero A., Prieto-Torres L. (2024). Tralokinumab-related facial redness: a therapeutic challenge. Int J Dermatol..

[2] Pastukhova E., Spurr A., Nakonechny Q., Lipson J. (2023). Upadacitinib-induced paradoxical face and scalp dermatitis: a case report of a novel sequela. Open Med Case Rep..

[3] Trave I., Salvi I., Canepa P., Parodi A., Cozzani E. (2024). Detection of demodex mites in papulopustular rosacea using microscopic examination and polymerase chain reaction: a comparative case-control study. Arch Dermatol Res..

[4] He W., Zheng C., Yang L., Tan J. (2025). Automatic fluorescence microscopic image analyzer: a novel AI-based tool for early diagnosing superficial fungal infections. BMC Infect Dis..

[5] Ahn J., Lee D.H., Na C.H., Shim D.H., Choi Y.S., Jung H.J. (2022). Facial erythema in patients with atopic dermatitis treated with Dupilumab – a descriptive study of morphology and Aetiology. J Eur Acad Dermatology Venereol..

[6] de Beer F.S.A., Bakker D.S., Haeck I., Ariens L., van der Schaft J., van Dijk M.R. (2019). Dupilumab facial redness: Positive effect of itraconazole. JAAD Case Rep..

[7] Chu H., Kim S.M., Zhang K.L., Wu Z., Lee H., Kim J.H. (2023). Head and neck dermatitis is exacerbated by Malassezia furfur colonization, skin barrier disruption, and immune dysregulation. Front Immunol..

[8] Zhu Gefei Alex, Chen Jennifer K., Chiou Albert, Justin Ko G.H. (2019). Assessment of the development of new regional dermatoses in patients treated for Atopic Dermatitis with dupilumab. J Am Acad Dermatol..

[9] Jo C.E., Finstad A., Georgakopoulos J.R., Piguet V., Yeung J., Drucker A.M. (2021). Facial and neck erythema associated with dupilumab treatment: a systematic review. J Am Acad Dermatol..

[10] Waldman R.A., DeWane M.E., Sloan B., Grant-Kels J.M. (2020). Characterizing dupilumab facial redness: a multi-institution retrospective medical record review. J Am Acad Dermatol..

[11] Seok S.H., An J.H., Shin J.U., Lee H.J., Kim D.H., Yoon M.S. (2020). Facial redness in atopic dermatitis patients treated with dupilumab: a case series. Allergy Asthma Immunol Res..

[12] Thyssen J.P. (2018). Could conjunctivitis in patients with atopic dermatitis treated with dupilumab be caused by colonization with demodex and increased interleukin-17 levels?. Br J Dermatol..

